# An In Vivo Study to Evaluate the Efficacy of Blue Shark (*Prionace glauca*) Cartilage Collagen as a Cosmetic

**DOI:** 10.3390/md20100633

**Published:** 2022-10-05

**Authors:** Wen-Chien Lu, Chien-Shan Chiu, Yung-Jia Chan, Tian-Pin Guo, Ching-Chin Lin, Po-Chun Wang, Po-Yu Lin, Amanda Tresiliana Mulio, Po-Hsien Li

**Affiliations:** 1 Department of Food and Beverage Management, Chung-Jen Junior College of Nursing, Health Sciences and Management, Chia-Yi City 60077, Taiwan; 2 Department of Dermatology, Taichung Veterans General Hospital, Taichung 40705, Taiwan; 3 College of Biotechnology and Bioresources, Da-Yeh University, Changhua 51591, Taiwan; 4 Pang-Ho Trading Co., Ltd., Tainan City 700020, Taiwan; 5 Wisdom International Pet Science Co., Ltd., Chiayi City 600051, Taiwan; 6 Department of Food and Nutrition, Providence University, Taichung City 43301, Taiwan

**Keywords:** *Prionace glauca*, shark cartilage, collagen type II, skin properties, cosmeceutical products

## Abstract

The “blue shark”, *Prionace glauca* (class: Chondrichthyes), is a pelagic shark species commonly found in tropical and temperate oceans. This shark is mainly sold in Asian countries as food and as traditional Chinese medicine. According to the Red List of the International Union for the Conservation of Nature, *P. glauca* is classified as low-risk to near endangered. *P. glauca* cartilage contains collagen type II, which makes it suitable as a bioactive ingredient in cosmeceutical products. This study evaluated the effects of a gel containing various concentrations (0.125–5%) of lyophilized hydrolyzed *P. glauca* cartilage on the human inner wrist skin compared to a placebo (base). A skin properties evaluation test was conducted before and after applying various concentrations (0.125–5%) of the *P. glauca* cartilage gel for 10 and 20 min on the inner wrists of participants using a skin analyzer that determined the moisture level, oil level, texture level, complexion level, and the 3D level. Adding lyophilized hydrolyzed shark cartilage (LHSC) significantly improved the moisture, texture, and complexion of the skin while controlling oil and providing a wrinkle-smoothing effect. The result indicated that LHSC formulations were prepared at different concentrations, and they had significantly enhanced effects on skin hydration and elasticity (texture) and the smoothing of wrinkles (3D level). The LHSC also effectively controlled oil secretion and the complexion.

## 1. Introduction

The blue shark, *Prionace glauca* (*P. glauca*) is the most abundant pelagic shark worldwide [1]. This shark species grows up to 380 cm in length and can live for 20 years. *P. glauca* is a cartilaginous fish in which no ossification occurs [2]; hence, the entire skeleton is composed of cartilage, which comprises 6% of its bodyweight [3]. *P. glauca* females are sexually mature at 5–6 years, whereas males mature at 4–5 years [4]. The shark’s meat and fins are targeted as a traditional food in Asian countries [5], and cartilage powder is used as a traditional Chinese medicine for arthritis and cancer treatment [6]. About 50–80% of fish waste, such as the skin, scales, bone, and viscera, is usually discarded immediately [7]. Recent studies have determined whether these unwanted products have value for further clinical use.

Collagen is an abundant fibrous structural protein in the extracellular matrix that accounts for up to 30% of total protein mass [8]. Collagen is found in the bones, joints, tendons, and skin [9]. Due to its triple helix molecular arrangement, it supports the body by providing stability and strength [10,11]. Due to its high biocompatibility and low immunogenicity, collagen is a superior ingredient in the cosmetic, food, and biomedical industries. Marine by-products, such as the skin, scales, bone, head, fins, and guts, which are low in value and cause enormous waste problems worldwide (20–80%), are promising sources for extracting collagen [12].

Mammalian collagen, such as bovine and porcine collagen, has been traditionally used before marine collagen. Bovine collagen from the bovine lung is the main source of collagen type I, while porcine collagen is extracted from porcine skin. These traditional sources of collagen are limited because of the risk of transmitting diseases, such as the flu and bovine spongiform encephalopathy, and because of restrictions by some religious groups [13]. Marine collagen has widely replaced mammalian collagen in cosmeceutical products and is considered safe. As a new alternative, marine collagen is in great demand because it is derived from fish bone and skin, which can be categorized as an atypical by-product [14].

*P. glauca* cartilage contains a high level of type II collagen; meanwhile, a high amount of type I collagen is also present in an adult shark’s vertebral cartilage. Type I collagen needs to achieve the moisturizing quality of marine collagen to be included as a component in cosmetics. The yields of raw *P. glauca* collagen under enzymatic extraction are 3.5% for pepsin-soluble collagen and 0.15% for acid-soluble collagen [15]. Collagen type II derived from shark cartilage is usually used to treat arthritis and other cartilage problems in humans but is not as popular as a bioactive ingredient in cosmetics. Instead, type II collagen is a product that mimics cartilaginous tissue [15] and is widely used in bone tissue engineering because it does not elicit an immune response [16]. Until now, capsules of shark cartilage have been marketed as an arthritis treatment, as it is a good source of chondroitin and glucosamine sulfate [17]. *P. glauca* skin contains collagen type I with a molecular weight of <20 KDa [18]. It is also valued as a material for cartilage tissue engineering. Yet, the effects of *P. glauca* cartilage as a bioactive ingredient in cosmeceutical products remain unknown.

Collagen has interesting biological activities, such as being antioxidant, anti-aging, moisturizing, anti-tumor, anti-microbial, an angiotensin-I inhibitor, and having wound-healing properties [19,20]. Hydrolyzed collagen is a harmless useful material [21]. Collagen can be extracted from marine by-products, and *P. glauca* cartilage was used in this study. Shark fins and meat are sold worldwide as traditional cuisine, and cartilage is used as a traditional Chinese medicine ingredient. Nevertheless, specific studies about the collagen extracted from *P. glauca* (blue shark) and its physicochemical properties are limited. This study investigated hydrolyzed *P. glauca* cartilage gel, which contains collagen type II, as a cosmeceutical product on human skin. The skin properties of moisture level, oil level, texture level, complexion level, and 3D level were studied for future applications in industry.

## 2. Results and Discussions

### 2.1. Physicochemical Properties of the Shark Cartilage

#### 2.1.1. Proximate Composition and Collagen

Table 1 shows the chemical compositions and yields of the hydrolyzed shark cartilage (HSC) and lyophilized hydrolyzed shark cartilage (LHSC). The average moisture content (%) of the HSC sample was 74.18 ± 0.26% of the total weight. Moisture content decreased to 15.41 ± 0.43% after freeze drying. The HSC samples had comparable moisture content with that of other shark species, such as *Carcharhinus albimarginatus* (63.56%) [22], *Chiloscyllium punctatum* (66.84%) [23], *Carcharhinus limbatus* (70.29%) [24], and *Isurus oxyrinchus* (78.30%) [25]. The ash contents of the HSC and LHSC samples were 5.13 ± 0.19% and 26.54 ± 0.24%, respectively. The LHSC had higher ash content than *Carcharhinus limbatus* (12.09%) [24], *Chiloscyllium punctatum* (15.79%) [24], *Carcharhinus albimarginatus* (25.41%) [22], and *Isurus oxyrinchus* (1.1%) [25]. The protein content of the HSC was 18.01 ± 0.15%, and that of the LHSC was 56.84 ± 0.21%. The protein content of brownbanded bamboo shark (*Chiloscyllium punctatum*) skin is 24.75% [23], and that of *P. glauca* from Vigo, Spain, is 54.44% [26]. The amino acid hydroxyproline, which is specific for collagen, was analyzed to determine collagen content. The hydroxyproline contents of the HSC and LHSC were 14.36 ± 0.23 and 27.10 ± 0.18 mg/g sample, respectively, which were higher than that of bigeye snapper bone (5.71 mg/g sample) [27]. Differences in species, seasonal variations, the environment, and body temperature could be why the hydroxyproline content varies among fish species [28]. The results showed that the samples used in this study were an excellent source of collagen.

#### 2.1.2. Color Analysis

**Figure 1:**
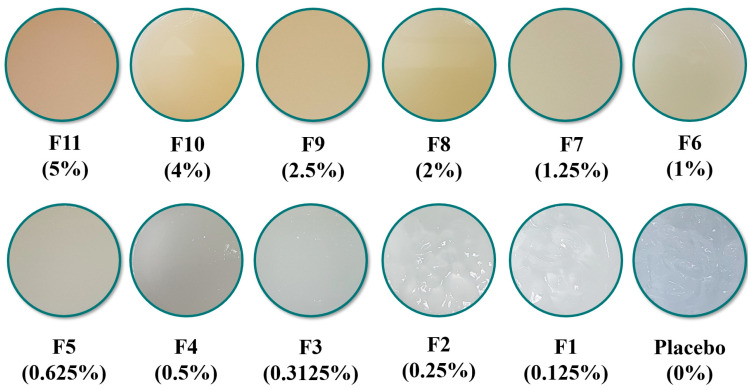
Different formulations of the lyophilized hydrolyzed shark cartilage (LHSC) gel.

Figure 1 and Table 2 present the color changes in hydrolyzed shark cartilage (HSC), lyophilized hydrolyzed shark cartilage (LHSC), and shark cartilage gel. The color of the liquid hydrolyzed *P. glauca* (HSC) was yellowish (L* = 5.43 ± 0.05; a* = −0.16 ± 0.14; b* = 21.07 ± 0.04). After being lyophilized, the liquid turned into a white solid mass form, it was spongy, and some parts turned into yellowish, transparent, thin layers. After being grounded, the LHSC was a sparkly white powder (L* = 53.87 ± 0.11; a* = −2.43 ± 0.08; b* = 8.45 ± 0.14) in color. Meanwhile, the prepared base was transparent, and the formulations were yellowish due to the presence of shark cartilage (Figure 1). The L* value decreased as the addition of LHSC increased. A higher concentration of shark cartilage caused changes in color, becoming more yellow and opaquer.

#### 2.1.3. Viscosity Test

Table 3 illustrates the results of the viscosity test for the control and the different concentrations of shark cartilage gel. In terms of the results, the sample gel developed lower viscosity as the concentration of shark cartilage increased (from 0.125% to 5%) and was unable to form a gel consistency at higher concentrations. The viscosity was 412.8 cP at the 5% concentration, whereas it was 4440.0 cP at the 0.125% concentration. Adding less LHSC resulted in a higher cP value, which would flow much slower. The tests revealed a relatively high variability in mechanical tensile resistance. The results demonstrated that the viscosity of the collagen solution was very sensitive to shear rate. Increasing the concentration of the collagen in a 0.1 M acetic aqueous solution (no salt) reduced the viscosity at the two different shear rates (25 °C); the reduced viscosity decreased with the increase in the shear rate, indicating a shear-thinning phenomenon [29]. Adsorption was not the only effect on viscosity behavior, as temperature and intermolecular, electrostatic forces caused differences in viscosity [29].

### 2.2. Dermatological Test

**Figure 2:**
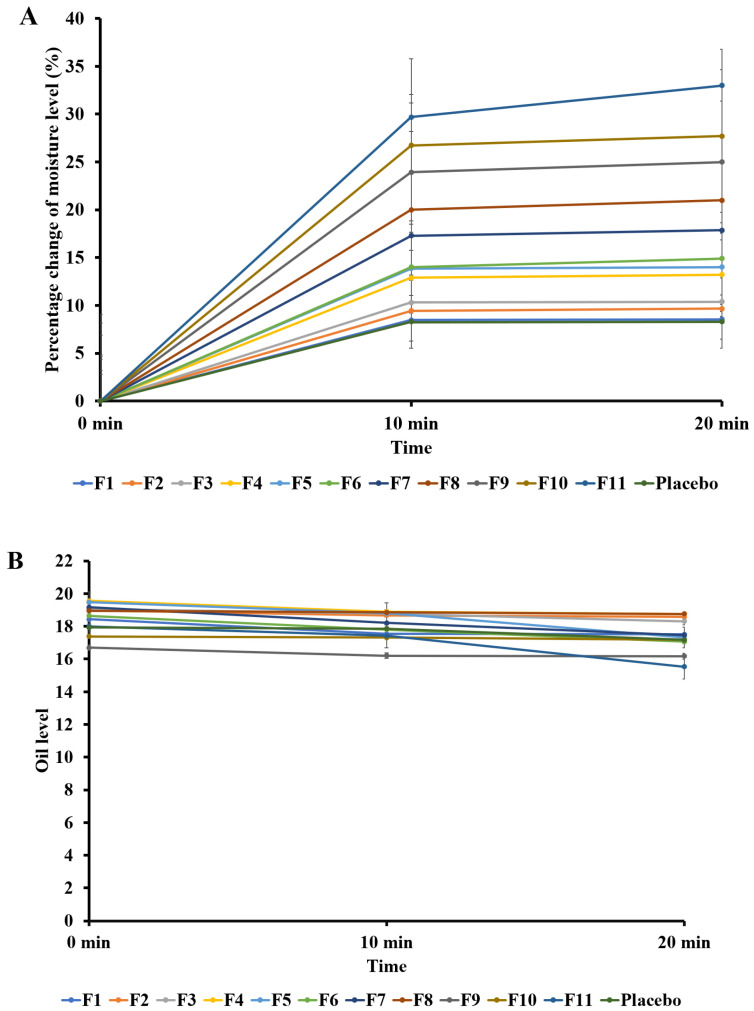

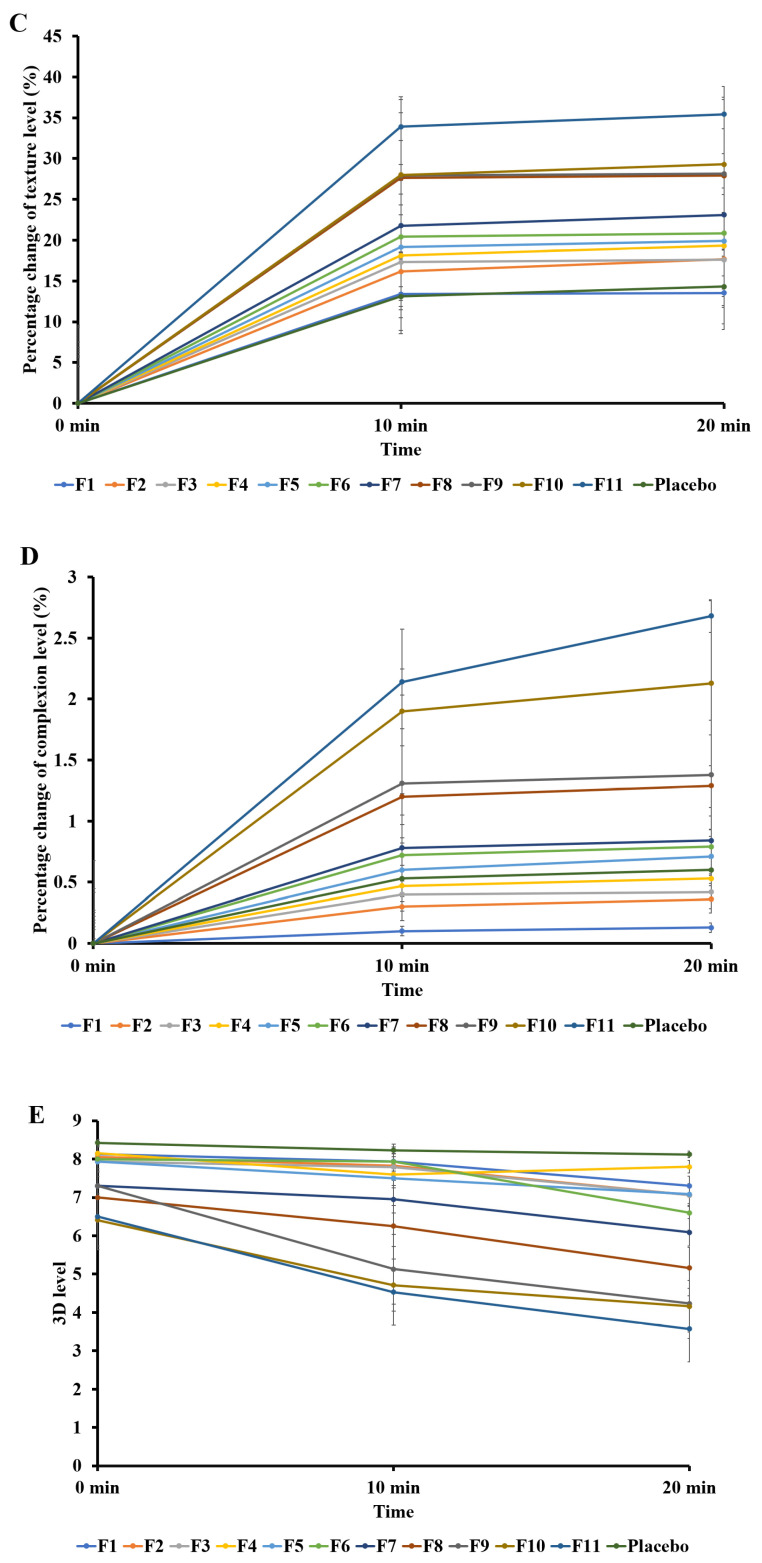
Dermatological analysis of the LHSC formulations in terms of (**A**) moisture level; (**B**) oil level; (**C**) texture level; (**D**) complexion level; and (**E**) the 3D level.

Figure 2 presents the skin properties’ analysis after the LHSC formulations were applied for 10–20 min. Figure 2A,C,D show upward trends in moisture, texture, and complexion, whereas oil secretion (Figure 2B) and the 3D level (Figure 2E) exhibited downward trends. Sample F11 improved the moisture, texture, and complexion of the skin. Sample F11 also significantly controlled oil secretion; meanwhile, the 3D structure was enhanced and the wrinkles smoothed after the F11 sample was applied for 10 and 20 min. Human skin is composed of epidermal and dermal layers. Collagen and elastin are present in the dermal layer to form the skin structure. The dermal layer receives fewer nutrients with age and aging damages the elasticity of the skin, as shown by the fragility of the collagen bundles [30]. Participants supplemented with 10 g of hydrolyzed *Pangasius hypophthalmus* collagen powder dissolved in 100 mL of water daily in the morning had improved wrinkle scores on both sides of the face and cheeks, improved skin hydration, and self-reported good elasticity, radiance, hydration, firmness, and wrinkle scores [30]. Due to the positive effect on the quality and functionality of the skin, it is possible to apply the cosmetic gel formulation with *P. glauca* collagen hydrolysate, which also indicates the biocompatibility with the skin and the biodegradability of the formulation.

The principle of moisture analysis is to determine the average moisture in the bottom skin layer. Skin is the largest and most important organ in the body as it accounts for 8% of the total weight of the body, accommodating approximately one-third of the circulating blood and one-quarter of the moisture in the body [31]. Skin aging is mainly determined by the moisture content in the skin, for example, water shortages in the skin results in dry skin and is harmful to sebaceous gland secretion, subcutaneous fat, and the elasticity of the tissue. The moisture content in the skin decreases under pathological conditions, such as skin inflammation, abnormal keratinocytes, eczema, dermatitis, and ichthyosis. Decreased moisture content in the stratum corneum can lead to skin dryness. Dry skin occurs due to genetics, environmental factors, and skin aging [32]. A deficiency in filaggrin units caused by a mutation in the gene is associated with dry skin. Environmental factors, such as low air humidity and over cleansing of the skin can also cause dry skin. The number of filaggrin units decreases in older people, which decreases the water-holding capacity. Different ethnicities also have differences in the frequency of dry skin [33].

Oil analysis was performed to analyze the excessive grease secretion from the skin. The lipids in the skin surface are composed of the sebum secreted by sebaceous glands and the epidermal lipids produced by disintegrating stratum corneum cells [34]. The sebum moves upward to the hair follicle sebaceous duct, and it is discharged at the surface of the skin. Keratin, which contains an acidic natural skin membrane, mainly consists of the sebaceous gland, and sweat gland secretions whose functions are to moisten the skin, reduce water loss, and prevent harmful material from damaging or invading the skin. Healthy skin is balanced between sebaceous secretions and moisture in the epidermal stratum corneum. The face feels greasy and not easy to clean when grease secretions are in excess. Some of the corresponding skin diseases that can occur are acne vulgaris and seborrheic dermatitis. Therefore, controlling these secretions from the sebum is the key to maintaining healthy and beautiful skin.

Texture analysis was carried out to analyze any texture disorders of the skin that occurred after applying the LHSC formulations. Dermatoglyphic, which is the study of the texture of the human skin, consists of the dermal furrow–hollow between the ridges and mammillary lines, which are in good order and parallel, formed by dermal papilla extruding from the epidermis. As age progresses, the dermal–epidermal junction becomes flat, the epidermis becomes thin, moisture volatilization increases fluid loss, epidermal cells develop poor metabolic status, the refresh rate is reduced, and the number of new cells decreases [35].

Skin color is determined by melanosomes, hemoglobin, carotenoids, dermis blood vessels, and dermis fibers, which change when they are exposed to ultraviolet light, medicines, and other irritants [36]. The difference in the upside composition of the stratum corneum is a vital factor in determining the skin surface reflection characteristics. The well-regulated reflection of the smooth stratum corneum with more moisture can form a bright luster, while the non-mirror reflection of the dry stratum corneum with scales reflects the light to make the skin gloomy. If the stratum corneum contains air, a white luster appears on the surface of the scales. Skin color is related to heredity but also the working environment and health status. Testing skin color and luster reflects the health level and the physiological and pathological changes causing inflammation. The lipids of the skin surface are composed of the sebum secreted by the sebaceous glands and the epidermal lipids produced by the disintegration of the stratum corneum cell. The generated sebum moves upward to the hair follicle’s sebaceous duct and is discharged through the surface of the skin.

*P. glauca* cartilage not only contained a high level of type II collagen, but also a high amount of type I collagen is also present in adult shark vertebral cartilage. Type I and type II collagen are the most common types found in cosmetics and supplements. Previous studies also concluded that marine-derived collagen extracted from salmon and codfish skin demonstrates a good capacity to retain water, thus being suitable for dermal applications as a moisturizer [37]. Except for its contributions as an anti-aging and anti-wrinkling product [38], collagen has long been used in the development of cosmetic formulations as a moisturizer and natural humectant [39]. Collagen is a natural skin moisturizer, as well as a natural humectant, and a film-forming substance. The ability of a film to form with collagen reduces transepidermal water loss [21]. Humectants act by binding water in the stratum corneum. Humectants were reported by Proksch et al. as one of five recommended key components in an ideal emollient [33]. According to their study, humectants are natural moisturizing factors that correct defects in skin elasticity. Humectants affect the organization of the lamellar lipids [33]. Cosmeceuticals are cosmetics combined with pharmaceuticals, which contain bioactive ingredients to improve skin conditions. Hydrolyzed collagen is a harmless and useful material [21]. Hydrolyzed collagen has good biological functions in cosmetics, such as anti-aging of the skin, retention, and increased cell proliferation [13]. This study demonstrated that LHSC provides another collagen source for cosmetic use.

## 3. Materials and Methods

### 3.1. Chemicals and Reagents

The chemical used to prepare the *P. glauca* gel included Carbopol (Lubrizol, Pharmaceuticals, Wickliffe, OH, USA), glycerin (Kao Corp., Tokyo, Japan), triethanolamine (TEA), an anti-microbial agent, and distilled water. All chemicals used in this study were American Chemical Society-certified grade and purchased from Sigma-Aldrich Chemical Co. (St. Louis, MO, USA).

### 3.2. Preparation of Shark Cartilage Extract

**Figure 3:**
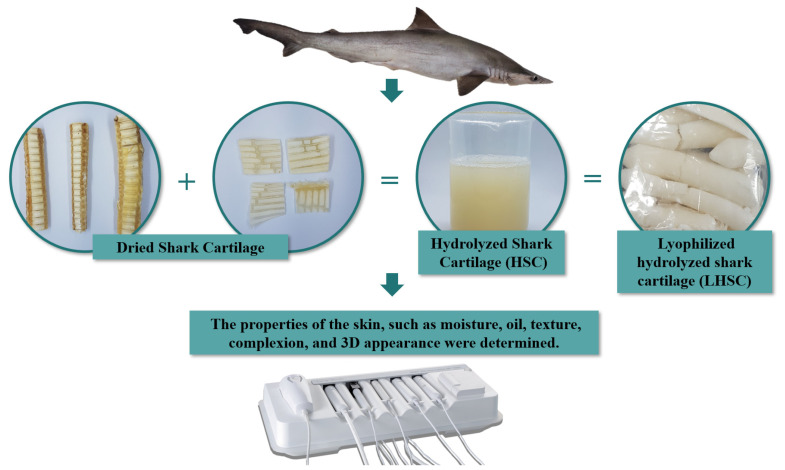
The experimental design was to test the effect of lyophilized hydrolyzed shark cartilage (LHSC) on skin health.

Figure 3 presents the experimental design for the effect of lyophilized hydrolyzed shark cartilage (LHSC) on skin health. Dried *P. glauca* cartilage was obtained from Pang-Ho Trading Co. The preparation of shark cartilage extract followed the method described by Li et al. [40] with modifications. The dried cartilage was washed with potable water and cut into small pieces before the collagen was isolated. The entire process of collagen isolation was performed at 4 °C. The cartilage was homogenized in a tissue homogenizer with phosphate buffer at pH 6.5 (0.2 mol/L sodium dihydrogen phosphate and 0.2 mol/L disodium hydrogen phosphate heptahydrate). The homogenate was treated with distilled water at a ratio of 1:5 (*w*/*v*) for 24 h to remove water-soluble substances. After centrifugation at 9000 rpm for 30 min, the pellet (cartilage samples) was decalcified with 0.3 M EDTA (pH 7.4) at a ratio of 1:10 (*w*/*v*) for 48 h. The solution was replaced every 12 h by centrifuging at 9000 rpm for 30 min to separate the supernatant and pellets. Next, the precipitate (pretreated shark cartilage) which was obtained by centrifugation at 9000 rpm for 30 min was soaked in 0.5 M acetic acid containing 1% pepsin (1:5, *w*/*v*) for 48 h with continuous shaking, and the extracts were centrifuged at 9000 rpm for 30 min at 4 °C. The supernatant was collected and salted out by adding 2 M sodium chloride (NaCl). The precipitate was obtained by centrifugation at 9000 rpm for 30 min. The precipitate was the hydrolyzed shark cartilage (HSC). The HSC crude collagen was then dissolved in 0.5 M acetic acid and dialyzed using a dialysis tube (Cellulose Membrane, D9777-100FT) with a typical molecular weight cut-off of 14 kDa, against 0.1 M acetic acid, followed by deionized water for 3 days. Before handling, the purified hydrolyzed *P. glauca* cartilage was stored at −20 °C. The HSC was weighed, the mass was recorded, and 30 mL was distributed to 50 mL falcon tubes; the samples were freeze-dried (FDM-5, UNISS, Taiwan) for 48 h. The LHSC was collected and weighed, and the percent yield was determined using the equation below. The lyophilized product was stored at −20 °C for further studies.

Yield (%) = (W_2_/W_1_) × 100% (1)

where W_1_ = is the weight of the dried shark cartilage, and W_2_ = is the weight of the shark cartilage powder after lyophilization.

### 3.3. Proximate Analysis

#### 3.3.1. Moisture Determination

An Association of Official Agricultural Chemists (AOAC) method was used with modifications. A clean glass crucible was placed in an oven at 105 °C for 1 h and transferred to a desiccator to cool down. The weight of the glass crucible was recorded. Then, 1 g of sample was spread evenly inside the glass crucible. The sample and the glass crucible were placed in a hot air oven at 135 °C for 2 h. The glass crucible was taken out and moved to a desiccator. The final weight of the glass crucible containing the dry sample was noted. Moisture content was determined using the following equation

Moisture (%) = [W_s_ − (W_2_ − W_1_)]/W_s_ × 100% (2)

where W_1_ = is the weight of the glass crucible; W_s_ = is the sample weight; and W_2_ is the weight of the glass crucible after drying.

#### 3.3.2. Ash Determination

An AOAC method was used with modifications. A clean crucible was put in a hot air oven at 105 °C for 20 min and cooled inside the desiccator. The weight of the blank crucible was recorded. Then, 1 g of sample was placed inside the crucible. The sample containing the crucible was placed in a muffle furnace at 600 °C for 12 h. The sample and crucible were weighed at room temperature, and the final weight of the crucible was noted. Ash content was determined by the equation

Ash (%) = (W_s_ − W_1_)/W_2_ × 100% (3)

where W_1_ = is the weight of the crucible; W_2_ = is the weight of the sample, and W_s_ is the weight of the crucible with ash.

#### 3.3.3. Hydroxyproline Content

The HSC and LHSC samples were hydrolyzed with 6 M HCl at 110 °C for 24 h. The hydrolysate was clarified with activated carbon and filtered through Whatman No. 4 filter paper. The filtrate was neutralized with 10 M and 1 M NaOH to obtain a pH of 6.0–6.5. The neutralized sample (0.1 mL) was transferred to a test tube and 0.2 mL of isopropanol was added and mixed well. Next, 0.1 mL of oxidant solution, which was a mixture of 7% (*w*/*v*) chloramine T and acetate/citrate buffer at pH 6, was added at a ratio of 1:4 (*v*/*v*) and mixed thoroughly. A 1.3 mL aliquot of Ehrlich’s reagent solution and isopropanol at a ratio of 3:13 (*v*/*v*) was added. The mixture was mixed and heated at 60 °C for 25 min in a water bath and then cooled for 2–3 min in running water. The solution was diluted to 5 mL with isopropanol. Absorbance was measured against water at a wavelength of 558 nm. Hydroxyproline standard solutions (10–80 ppm) were included. Hydroxyproline content was calculated and expressed as mg/g sample.

### 3.4. Preparation of the Topical Formulation

The gel base was prepared as the placebo by swelling Carbopol in hot water for 24 h. TEA was added to the swelled Carbopol solution and stirred. After the gel base was formed, glycerin, the remaining water, and an anti-microbial agent were added, and the solution was mixed until homogenous. The shark cartilage formulation was prepared using the same method, except various concentrations of freeze-dried shark cartilage diluted in hot water with glycerin and an anti-microbial agent was added and mixed until homogenous (Table 4).

### 3.5. Parameters for Evaluating the Formulation Characteristics

#### 3.5.1. Color Test

The color of the LHSC sample powder and the formulated gel samples were analyzed with the Color Meter ZE-2000 (Nippon Denshku Industries Co., Ltd., Tokyo, Japan). The L* (lightness measurement), a* (greenness–redness value), and b* (blueness–yellowness value) values of the samples were determined to analyze changes in color quality. The instrument was calibrated with a standard black-and-white ceramic tile before measurements. The color measurements were performed in triplicate at room temperature.

#### 3.5.2. Viscosity Test

The viscosity of the prepared base and the formulations were determined with a Brookfield DV-E Viscometer. The viscosities of the base and the formulations were measured with the RPM 100 using spindle number 64. Each of the formulations was tested immediately after removal from a 6 °C refrigerator because viscosity is affected by temperature.

### 3.6. Dermatological Test

**Figure 4:**
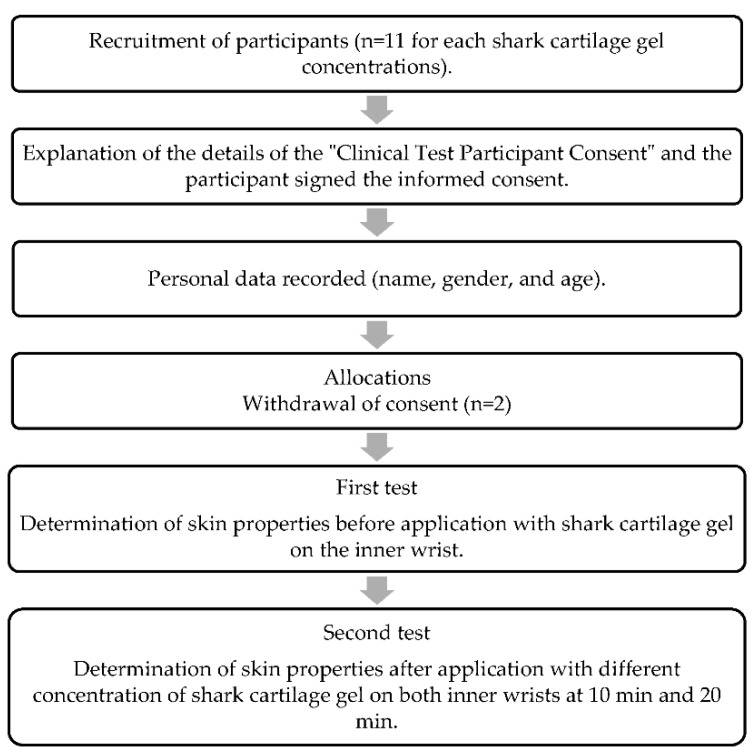
In vivo study protocol to investigate the effects of the LHSC formulations.

Participants that met the criteria for the experiment were selected through interview. All participants were verbally informed about the study. Participants who had a skin allergic reaction within 30 days did not participate in this dermatological test. Eleven participants were selected for each formulation (Figure 4). Two formulations were tested per volunteer with one formulation on each inner wrist. In total, 66 volunteers (33 females and 33 males, age range 18–25 years) participated in this experiment. The participants were students at Dayeh University, Dacun, Changhua, Taiwan. The properties of the inner wrist skin, such as moisture, oil, texture, complexion, and 3D appearance were determined before applying the prepared base and formulations and 10 min and 20 min after applying the formulations using Multi Skin Test Center MC 1000 (Courage-Khazaka Electronic GmbH, Cologne, Germany). The participants were preconditioned in the test room for at least 15 min before the measurements.

The equation used to measure the percent change in the skin parameters of the volunteers was

Percentage change = [(N_x_ − N_0_)/N_0_] × 100% (4)

N_x_ is the value of the parameter during the first 10 and 20 min, and N_0_ = is the zero-hour value of the parameter.

### 3.7. Ethical Approval and Informed Consent

This study protocol followed Good Clinical Practices and the Declaration of Helsinki and agreed with the appropriate institutional review board (IRB) regulations. The experimental protocol was registered at the Taichung Jen Ai Hospital. This protocol was approved by the IRB of Taichung Jen Ai Hospital, Taichung, Taiwan (clinical trial approval certificate no. 110-12). The participants gave informed consent before beginning any study-related procedures or medications.

### 3.8. Statistical Analysis

Data are presented as mean ± SD. Differences were detected using the paired Student’s *t*-test and SPSS 15.0 software (SPSS Inc., Chicago, IL, USA). A *p*-value < 0.05 was considered significant.

## 4. Conclusions

*P. glauca* is the most abundant pelagic shark worldwide. In this study, dried *P. glauca* cartilage was used to extract the hydrolyzed shark cartilage. LHSC formulations were prepared at different concentrations; the L* value was decreased as the addition of LHSC increased. Higher concentrations of shark cartilage caused changes in color, becoming more yellow and opaquer. Moreover, LHSC formulations tended to enhance skin hydration and elasticity (texture) and smooth wrinkles (3D level). The LHSC also effectively controlled oil secretion and the complexion.

## Figures and Tables

**Table 1:** Chemical compositions and yields of the hydrolyzed shark cartilage (HSC) and the lyophilized hydrolyzed shark cartilage (LHSC).

Sample	Proximate Composition (%)			Hydroxyproline Content (mg/g)	Yield (%)
	Moisture	Ash	Protein		
HSC	74.18 ± 0.26 ^a^	5.13 ± 0.19 ^b^	18.01 ± 0.15 ^b^	14.36 ± 0.23 ^b^	-
LHSC	15.41 ± 0.43 ^b^	26.54 ± 0.24 ^a^	56.84 ± 0.21 ^a^	27.10 ± 0.18 ^a^	3.09 ± 0.07

Different superscript lowercase letters in the same column indicate significant differences (*p* < 0.05). All data are mean ± SD.

**Table 2:** Color changes in hydrolyzed shark cartilage (HSC), lyophilized hydrolyzed shark cartilage (LHSC), and shark cartilage gel.

Sample	L*	a*	b*	ΔE
HSC	5.43 ± 0.05 ^h^	−0.16 ± 0.14 ^b^	21.07 ± 0.04 ^a^	0.11 ± 0.08 ^de^
LHSC	53.87 ± 0.11 ^d^	−2.43 ± 0.08 ^g^	8.45 ± 0.14 ^cd^	0.18 ± 0.13 ^d^
F1	79.30 ± 0.19 ^b^	−0.08 ± 0.09 ^a^	3.88 ± 0.06 ^ef^	0.24 ± 0.21 ^c^
F2	79.10 ± 0.15 ^b^	−0.12 ± 0.09 ^ab^	5.80 ± 0.10 ^e^	0.32 ± 0.22 ^b^
F3	77.83 ± 0.18 ^b^	−0.13 ± 0.16 ^ab^	6.08 ± 0.09 ^d^	0.19 ± 0.19 ^d^
F4	67.10 ± 0.12 ^c^	−0.20 ± 0.10 ^c^	7.07 ± 0.06 ^d^	0.24 ± 0.11 ^c^
F5	65.70 ± 0.06 ^c^	0.22 ± 0.06 ^c^	8.64 ± 0.03 ^cd^	0.11 ± 0.10 ^de^
F6	38.21 ± 0.05 ^e^	0.29 ± 0.04 ^d^	9.31 ± 0.11 ^c^	0.37 ± 0.17 ^b^
F7	38.04 ± 0.04 ^e^	0.32 ± 0.14 ^d^	9.40 ± 0.10 ^c^	0.24 ± 0.12 ^c^
F8	18.91 ± 0.14 ^f^	−0.38 ± 0.60 ^e^	11.13 ± 0.16 ^b^	0.26 ± 0.14 ^c^
F9	15.32 ± 0.11 ^f^	0.39 ± 0.22 ^e^	12.04 ± 0.12 ^b^	0.32 ± 0.11 ^b^
F10	12.61 ± 0.15 ^fg^	−0.46 ± 0.17 ^f^	13.09 ± 0.17 ^b^	0.56 ± 0.03 ^a^
F11	10.50 ± 0.06 ^g^	−0.50 ± 0.13 ^f^	13.40 ± 0.13 ^b^	0.10 ± 0.08 ^de^
Placebo	89.00 ± 0.13 ^a^	−0.37 ± 0.16 ^e^	1.77 ± 0.06 ^f^	0.22 ± 0.11 ^c^

Different superscript lowercase letters in the same column indicate significant differences (*p* < 0.05). All data are mean ± SD.

**Table 3:** Results of the viscosity test for the prepared base (placebo) and the shark cartilage gel.

Formulations	Viscosity (cP)
F1	4440.0 ± 0.05 ^b^
F2	4374.0 ± 0.13 ^b^
F3	3220.8 ± 0.11 ^c^
F4	2904.0 ± 0.08 ^d^
F5	1192.8 ± 0.14 ^e^
F6	808.8 ± 0.21 ^f^
F7	470.4 ± 0.18 ^g^
F8	379.6 ± 0.14 ^h^
F9	222.6 ± 0.09 ^i^
F10	180.0 ± 0.04 ^j^
F11	152.8 ± 0.12 ^j^
Placebo	5925.6 ± 0.11 ^a^

Different superscript lowercase letters in the same column indicate significant differences (*p* < 0.05). All data are mean ± SD.

**Table 4:** Composition of formulations.

Formula	Composition %					
	Water	LHSC Powder *	Carbopol	TEA	Glycerin	Anti-microbial Agent
F1	100	0.125	0.2	0.2	5	0.3
F2	100	0.25	0.2	0.2	5	0.3
F3	100	0.3125	0.2	0.2	5	0.3
F4	100	0.5	0.2	0.2	5	0.3
F5	100	0.625	0.2	0.2	5	0.3
F6	100	1	0.2	0.2	5	0.3
F7	100	1.25	0.2	0.2	5	0.3
F8	100	2	0.2	0.2	5	0.3
F9	100	2.5	0.2	0.2	5	0.3
F10	100	4	0.2	0.2	5	0.3
F11	100	5	0.2	0.2	5	0.3
Placebo	100	0	0.2	0.2	5	0.3

* LHSC powder referred to the lyophilized hydrolyzed shark cartilage powder.

## Data Availability

Data sharing not applicable. No new data were created or analyzed in this study. Data sharing is not applicable to this article.

## References

[1] Veríssimo A., Sampaio Í., McDowell J.R., Alexandrino P., Mucientes G., Queiroz N., da Silva C., Jones C.S., Noble L.R. (2017). World without borders—Genetic population structure of a highly migratory marine predator, the blue shark (*Prionace glauca*). Ecol. Evol..

[2] Merly L., Smith S.L. (2013). Collagen type II, alpha 1 protein: A bioactive component of shark cartilage. Int. Immunopharmacol..

[3] Burnsed O.A., Schwartz Z., Marchand K.O., Hyzy S.L., Olivares-Navarrete R., Boyan B.D. (2016). Hydrogels derived from cartilage matrices promote induction of human mesenchymal stem cell chondrogenic differentiation. Acta Biomater..

[4] Biton-Porsmoguer S., Bǎnaru D., Boudouresque C.F., Dekeyser I., Bouchoucha M., Marco-Miralles F., Lebreton B., Guillou G., Harmelin-Vivien M. (2018). Mercury in blue shark (*Prionace glauca*) and shortfin mako (*Isurus oxyrinchus*) from north-eastern Atlantic: Implication for fishery management. Mar. Pollut. Bull..

[5] Kim S.W., Han S.J., Kim Y., Jun J.W., Giri S.S., Chi C., Yun S., Kim H.J., Kim S.G., Kang J.W. (2019). Heavy metal accumulation in and food safety of shark meat from Jeju Island, Republic of Korea. PLoS ONE.

[6] Still J. (2003). Use of animal products in traditional Chinese medicine: Environmental impact and health hazards. Complement. Ther. Med..

[7] Arvanitoyannis I.S., Kassaveti A. (2008). Fish industry waste: Treatments, environmental impacts, current and potential uses. Int. J. Food Sci. Technol..

[8] Sun B. (2021). The mechanics of fibrillar collagen extracellular matrix. Cell Rep. Phys. Sci..

[9] Avila Rodríguez M.I., Rodríguez Barroso L.G., Sánchez M.L. (2018). Collagen: A review on its sources and potential cosmetic applications. J. Cosmet. Dermatol..

[10] Kirkness M.W., Lehmann K., Forde N.R. (2019). Mechanics and structural stability of the collagen triple helix. Curr. Opin. Chem. Biol..

[11] Shoulders M.D., Raines R.T. (2009). Collagen structure and stability. Annu. Rev. Biochem..

[12] Renuka V., Remya S., Jha A., Joseph T. (2019). Nature and Composition of Fish Processing Industrial Waste and Handling Protocols.

[13] León-López A., Morales-Peñaloza A., Martínez-Juárez V.M., Vargas-Torres A., Zeugolis D.I., Aguirre-Álvarez G. (2019). Hydrolyzed collagen—Sources and applications. Molecules.

[14] Liu J., Shibata M., Ma Q., Liu F., Lu Q., Shan Q., Hagiwara T., Bao J. (2020). Characterization of fish collagen from blue shark skin and its application for chitosan-collagen composite coating to preserve red porgy (*Pagrus major*) meat. J. Food Biochem..

[15] Seixas M.J., Martins E., Reis R.L., Silva T.H. (2020). Extraction and characterization of collagen from elasmobranch byproducts for potential biomaterial use. Mar. Drugs.

[16] Elango J., Lee J.W., Wang S., Henrotin Y., De Val J.E.M.S., Regenstein J.M., Lim S.Y., Bao B., Wu W. (2018). Evaluation of differentiated bone cells proliferation by blue shark skin collagen via biochemical for bone tissue engineering. Mar. Drugs.

[17] Musick J.A., Bonfil R. (2005). Management Techniques for Elasmobranch Fisheries.

[18] Sanchez A., Blanco M., Correa B., Perez-Martin R.I., Sotelo C.G. (2018). Effect of fish collagen hydrolysates on type I collagen mRNA levels of human dermal fibroblast culture. Mar. Drugs.

[19] Ahmed M., Verma A.K., Patel R. (2020). Collagen extraction and recent biological activities of collagen peptides derived from sea-food waste: A review. Sustain. Chem. Pharm..

[20] Felician F.F., Xia C., Qi W., Xu H. (2018). Collagen from marine biological sources and medical applications. Chem. Biodivers..

[21] Sionkowska A., Adamiak K., Musiał K., Gadomska M. (2020). Collagen based materials in cosmetic applications: A review. Materials.

[22] Jeevithan E., Bao B., Bu Y., Zhou Y., Zhao Q., Wu W. (2014). Type II collagen and gelatin from silvertip shark (*Carcharhinus albimarginatus*) cartilage: Isolation, purification, physicochemical and antioxidant properties. Mar. Drugs.

[23] Kittiphattanabawon P., Benjakul S., Visessanguan W., Kishimura H., Shahidi F. (2010). Isolation and characterisation of collagen from the skin of brownbanded bamboo shark (*Chiloscyllium punctatum*). Food Chem..

[24] Kittiphattanabawon P., Benjakul S., Visessanguan W., Shahidi F. (2010). Isolation and characterization of collagen from the cartilages of brownbanded bamboo shark (*Chiloscyllium punctatum*) and blacktip shark (*Carcharhinus limbatus*). LWT-Food Sci. Technol..

[25] Cho S., Kwak K., Park D., Gu Y., Ji C., Jang D., Lee Y., Kim S. (2004). Processing optimization and functional properties of gelatin from shark (*Isurus oxyrinchus*) cartilage. Food Hydrocoll..

[26] Vázquez J.A., Blanco M., Fraguas J., Pastrana L., Pérez-Martín R. (2016). Optimisation of the extraction and purification of chondroitin sulphate from head by-products of Prionace glauca by environmental friendly processes. Food Chem..

[27] Kittiphattanabawon P., Benjakul S., Visessanguan W., Nagai T., Tanaka M. (2005). Characterisation of acid-soluble collagen from skin and bone of bigeye snapper (*Priacanthus tayenus*). Food Chem..

[28] Elango J., Bu Y., Bin B., Geevaretnam J., Robinson J.S., Wu W. (2017). Effect of chemical and biological cross-linkers on mechanical and functional properties of shark catfish skin collagen films. Food Biosci..

[29] Li Y., Qiao C., Shi L., Jiang Q., Li T. (2014). Viscosity of collagen solutions: Influence of concentration, temperature, adsorption, and role of intermolecular interactions. J. Macromol. Sci. Part B.

[30] Evans M., Lewis E.D., Zakaria N., Pelipyagina T., Guthrie N. (2021). A randomized, triple-blind, placebo-controlled, parallel study to evaluate the efficacy of a freshwater marine collagen on skin wrinkles and elasticity. J. Cosmet. Dermatol..

[31] Toriyama M., Ishii K.J. (2021). Primary cilia in the skin: Functions in immunity and therapeutic potential. Front. Cell Dev. Biol..

[32] De Miranda R.B., Weimer P., Rossi R.C. (2021). Effects of hydrolyzed collagen supplementation on skin aging: A systematic review and meta-analysis. Int. J. Dermatol..

[33] Proksch E., Berardesca E., Misery L., Engblom J., Bouwstra J. (2020). Dry skin management: Practical approach in light of latest research on skin structure and function. J. Dermatol. Treat..

[34] Caliskan U.K., Karakus M.M. (2020). Essential oils as skin permeation boosters and their predicted effect mechanisms. J. Dermatol. Ski. Sci..

[35] Bogdan C., Iurian S., Tomuta I., Moldovan M. (2017). Improvement of skin condition in striae distensae: Development, characterization and clinical efficacy of a cosmetic product containing Punica granatum seed oil and Croton lechleri resin extract. Drug Des. Dev. Ther..

[36] Tsumura N., Ojima N., Sato K., Shiraishi M., Shimizu H., Nabeshima H., Akazaki S., Hori K., Miyake Y. (2003). Image-based skin color and texture analysis/synthesis by extracting hemoglobin and melanin information in the skin. ACM Trans. Graph..

[37] Alves A.L., Marques A.L., Martins E., Silva T.H., Reis R.L. (2017). Cosmetic potential of marine fish skin collagen. Cosmetics.

[38] Xhauflaire-Uhoda E., Fontaine K., Piérard G. (2008). Kinetics of moisturizing and firming effects of cosmetic formulations. Int. J. Cosmet. Sci..

[39] Peng Y., Glattauer V., Werkmeister J.A., Ramshaw J.A. (2004). Evaluation for collagen products for cosmetic application. Int. J. Cosmet. Sci..

[40] Li P.-H., Lu W.-C., Chan Y.-J., Ko W.-C., Jung C.-C., Le Huynh D.T., Ji Y.-X. (2020). Extraction and characterization of collagen from sea cucumber (*Holothuria cinerascens*) and its potential application in moisturizing cosmetics. Aquaculture.

